# Design and Control of Magnetic Shape Memory Alloy Actuators

**DOI:** 10.3390/ma15134400

**Published:** 2022-06-22

**Authors:** Bartosz Minorowicz, Andrzej Milecki

**Affiliations:** Faculty of Civil and Transport Engineering, Poznan University of Technology, Piotrowo Street 3, 60-965 Poznan, Poland; andrzej.milecki@put.poznan.pl

**Keywords:** shape memory alloys, MSMA, actuator design, smart materials, hysteresis, modelling

## Abstract

This paper presents research on the application of magnetic shape memory alloys (MSMAs) in actuator design. MSMAs are a relatively new group of so-called smart materials that are distinguished by repeatable strains up to 6% and dynamics much better than that of thermally activated shape memory alloys (SMAs). The shape change mechanism in MSMAs is based on the rearrangement of martensite cells in the presence of an external magnetic field. In the first part of the article a review of the current state of MSMA actuator design is presented, followed by a description of the design, modelling and control of a newly proposed actuator. The developed actuator works with MSMA samples of 3 × 10 × 32 mm^3^, guaranteeing an available operating range of up to 1 mm, despite its great deformation range and dynamics. In the paper its dynamics model is proposed and its transfer function is derived. Moreover, the generalised Prandtl-Ishlinskii model of MSMA-actuator hysteresis is proposed. This model is then inverted and used in the control system for hysteresis compensation. A special test stand was designed and built to test the MSMA actuator with compensation. The step responses are recorded, showing that the compensated MSMA actuator exhibits the positioning accuracy as ±2 µm. As a result, the authors decided to apply a control system based on an inverse hysteresis model. The paper concludes with a summary of the research results, with theoretical analysis compared with the registered actuator characteristics.

## 1. Introduction

### 1.1. Preface

In the last three decades, the number of investigations focusing on the application of a new type of drive system known as unconventional, smart, or even intelligent has increased significantly, typically with the aim of meeting new engineering requirements such as better dynamic performance, an increase in the power to weight ratio, weight reduction, and the improvement of positioning accuracy and durability. New, advanced drives are especially in demand for use in high precision positioning devices, special machines, precise manipulators, medicinal devices and military equipment [1], with their construction frequently involving the use of so-called smart materials to replace complex mechanical structures such as torque motors in servo valves. Studies examining the development of new actuators based on smart materials have been undertaken at both academic institutions [2,3] and companies, e.g., ETO Group [4].

### 1.2. Smart Materials

Smart materials are now most commonly used as drive elements. For example, piezoelectric crystals have been employed in engineering applications since the mid-19th century and are currently used in high-precision actuators, such as those of atomic force microscopes [5]. Such crystals are distinguished by their high dynamic bandwidth, sub-nanometre resolution and very short response times, making them suitable for fast and very accurate positioning tasks. One interesting application of piezo elements is their use in pneumatic or hydraulic servo-valves, where a very precise torque motor is replaced by piezo stacks [6,7] or piezo bender actuators [8,9]. Other smart materials used today in precise drives may be magnetically active, including magnetostrictive materials such as Terfenol-D. Although both piezo and magnetostrictive elements are characterised by their high dynamics, they exhibit rather weak deformation of not more than tens of micrometres [10,11]. Shape memory alloys (SMAs), initially developed in the mid-20th century, are currently attracting increased attention in a wide range of disciplines as a competitive alternative to other non-classical actuators. The main distinguishing feature of SMAs is their large deformation in comparison to other groups of smart materials. Unfortunately, the dynamics of thermally activated SMAs (TSMAs) is very weak, particularly during the cooling phase, with time constants of step responses equal to a few seconds [12,13]. Nevertheless, interesting applications of TSMAs include their use in bi-stable safety valves, which open automatically with a rise in ambient temperature (thermostat mode). In such instruments the TSMA wires work without an electronic control system, acting as a safety valve [14]. The design and nonlinear control of beam-like structural deflection by TSMA wires is presented in papers [12,15,16]. Technical analysis of the practical application of SMA structures is presented in paper [2], which provides a review of recent research and the commercial application of SMAs, including one hundred patents categorised against relevant commercial domains and rated with respect to relevant design objectives.

One special group of SMAs are the magnetic shape memory alloys (MSMAs), active materials that change properties such as their shape and relative magnetic permeability in the presence of an external magnetic field, generating force and repeatable strains of up to 6%. The first mention of crystal lattice reorientation was published by Ullakko in 1996 [17,18]. The main motivation behind the design of MSMA-based actuators is to fill the gap between actuators based on TSMAs and those built of magnetostrictive materials, combining the strain scope of the former and a dynamic response expressed in milliseconds comparable to that of the latter [19].

As every application of MSMAs requires the generation of a magnetic field, the key element in MSMA-based drives is the magnetic circuit and a coil, the design of which is very important. Although the German company ETO Magnetic reported [20] that excitation by large currents provided very satisfying results (short response times), but the high current density in the coil increased the temperature of the actuator, which represents a considerable disadvantage because MSMAs are very sensitive to temperature changes [21]. In contrast to conventional heat-driven shape memory alloys, MSMAs exhibit higher operating frequencies, making them attractive for many actuation applications. Indeed, such properties are highly promising regarding the use of MSMAs in low displacement positioning devices. Practically, MSMAs are currently the only smart material offering the possibility of generating strains of up to 1000 µm with dynamics significantly better than that of TSMAs. To obtain a strain of about 100 µm, actuators constructed of piezo or magnetostrictive materials require mechanical amplification, for example by a complex lever system. Such a solution complicates the structure of the actuator, and reduces both its accuracy and dynamics. Mechanical amplification also provides a relatively small increase in strain, for example the strain of the actuator presented in [6] was increased only three-fold (from 30 µm to 90 µm).

Most actuators based on smart materials are as yet not commercial products due to their nonlinear behaviour, which makes it difficult to regulate position via open and closed loop control. The most important of these characteristics is hysteresis, which significantly influences positioning accuracy and thus many efforts have been made to overcome this problem. One of the most popular strategies involves initial hysteresis modelling, then inversing the model and finally applying it as the compensator in an open or closed loop control system [12,22]. Although it is possible to control an actuator with hysteresis via a closed-loop system without hysteresis compensation, this method is much more difficult and can cause drive inaccuracy and instability, with the actuator also tending to fall into oscillatory behaviour [23].

The modelling of hysteresis strongly depends on its shape (with or without saturation, symmetrical or not-symmetrical, sharp); for example, the model used for the compensation of hysteresis characterised by symmetric loops without saturation cannot be satisfactorily applied to compensate other hysteresis shapes. Hysteresis modelling can be split into two main techniques, the first based on the mathematical description of physical phenomena and the second based on phenomenological theory. Among these models, the generalised Prandtl-Ishlinskii model (GPI) appears to be highly promising due to its ability to model different hysteresis shapes and the fact that it can be quite easily analytically inverted [23].

MSMA actuators are still relatively new and are not yet ready for commercial application [4]. Indeed, there are currently no detailed publications available in the literature regarding the design of actuators using MSMA materials. Therefore, the present paper provides a short description of the MSMA design process, with a particular focus on magnetic circuits and mechanical structure, assuming that the designed actuator should be able to provide an operating range of up to 1 mm. The second contribution of this paper is hysteresis modelling and compensation using the generalised Prandtl-Ishlinskii model, thus enabling the linearization of input-output characteristics. In the following step, the hysteresis model is expanded via the addition of a dynamic actuator model, with control methods for the designed and built actuator then described. The results of experimental investigations carried out using a prepared test stand are then described for different control strategies. The structure of the paper is arranged as follows: the paper starts with a brief overview of MSMAs and a review of the current state of MSMA actuators, followed by an outline of the actuator design process and the modelling of MSMA static and dynamic characteristics. In the next section, hysteresis compensation using an inverse phenomenological model is described. Finally, the results of experimental investigation into the MSMA-based actuator with hysteresis compensation and PID regulator control are presented, and conclusions discussed.

In this paper the investigations performed by the authors have also been included. The main research objective is to improve the MSMA actuator performance, especially its positioning accuracy. The second objective is to propose the theoretical description of the actuator. Two actuators based on different MSMA pieces are designed and built. Some useable equations in the design of the magnetic circuit and the actuator’s are given. The test stand and the basic investigation results of the MSMA are described. The important contribution of the article is to propose the inverse of the generalised Prandtl-Ishlinskii (GP-I) model of MSMA actuator. This model is used for successful compensation of the actuator hysteresis. Compared to existing works, this approach is relatively new. The developed inverse (GP-I) model is used for MSMA actuator’s positioning accuracy improvement.

## 2. Magnetic Shape Memory Alloys and Their Use as Actuators

### 2.1. Basics of MSMA—Reorientation of a Crystal Lattice

The magnetic shape memory effect is a result of the cell reorientation at low temperatures (below 60 °C) of a specially prepared crystal lattice. At such temperatures, the material is in a martensitic state and consists of cuboidal cells. For example, Ni_2_MnGa MSMA comprises cells with dimensions *a* = *b* = 0.594 nm, *c* = 0.562 nm. These cells can be set in three possible directions known as variants *V*_1_, *V*_2_ and *V*_3_. The magnetic permeability of the cells is different in each direction, with the largest value being in the axis parallel to the direction of side *c* and perpendicular to the cell surface indicated by edges *a* and *b*, also known as the easy magnetisation axis. In the initial phase, all cells in the MSMA probe are in variant *V*_1_; the probe length is *L_z_*. When a magnetic field of strength *H_ext_* is applied to an MSMA, the cuboidal cells tend to move to set their easy magnetisation axis in the same direction as the magnetic field direction i.e., in variant *V*_2_. Furthermore, when the energy needed for cell rotation is greater than that needed for twin boundary motion, the magnetisation process causes rearrangements in the crystal lattice. As a result, the volumes of variants *V*_1_ and *V*_2_ change, which is visible as a difference in shape, and thus the material changes its length in a direction perpendicular to magnetic field lines and can be used for force generation and position regulation. The most commonly used material in MSMA actuators is Ni_2_MnGa, which offers a repeatable scope of elongation of up to 6% of the initial length.

**Figure 1:**
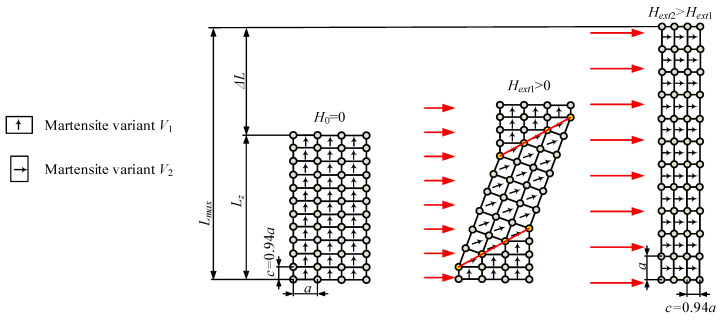
Deformation of a crystal lattice in a magnetic field, where *a* and *c* are the lengths of a single martensite cell (*c =* 0.94*a*).

The return transformation is possible when the magnetic field is switched off and a compressive force applied. Thus, the subsequent stress in the material favours the growth of martensite variants whose shorter side is parallel to the direction of the compressive force, with the structure of the material during the squeezing phase transforming into a single variant structure (e.g., *V*_1_). Because the dimensions of the martensite cell variants are different, after reorientation the MSMA shortens in length. The above-described process is presented schematically in Figure 1.

Generally, it is possible to obtain complex deformation in 3 directions, with produced samples formed in such a way that ensures that the result of deformation will be useful and can generate movement. Referring to Figure 1, the calculation of theoretical maximum probe length is possible using the following equation [24]:

(1) $$L_{\max}=L_{z}\cdot (1+\varepsilon_{0})$$

where *L*_max_ is the maximum length of the MSMA material, *L_z_* is the length of the MSMA before deformation and *ε*_0_ is the dimensionless free strain expressed as 1–*c*/*a*. 

### 2.2. MSMA Actuator Design Survey

According to the literature [25,26] there are five basic operating modes for magnetic shape memory alloys, the most common of which is so-called ‘spring returned’ mode [27,28,29,30]. Other known designs include push—push and push—pull, which are effectively different names for the same operating idea [31,32,33].

**Figure 2:**
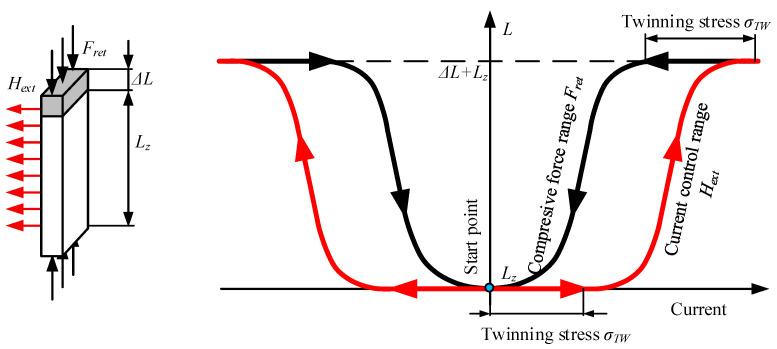
Spring operating mode—theoretical curve strain vs. current, where *L_z_* is the length of the MSMA element, ∆*L* is MSMA deformation, *H_ext_* is the strength of the external magnetic field, *F_ret_* is the compressive force, and *σ_TW_* is the twinning stress.

Spring mode (Figure 2) is most suitable for use in devices whose major task is precise micro positioning. The volume of reoriented martensite is proportional to the strength of the applied magnetic field (mostly generated by coils), with MSMA probe deformation observed at the macroscopic scale as a strain. The magnetic shape memory effect itself is self-supporting; only the application of an external force which results in material stress greater than the twinning stress *σ_TW_* can restore the original length. In practice (i.e., the easiest method of application) this force is produced by a compression coil spring, but the compressive force is not sufficient to return the sample to its original shape after the first cycle of operation. This represents a distinct disadvantage, as it reduces the maximum scope of strain in subsequent cycles and increases the movable mass.

From a mechanical point of view, the most important design issue is the assurance of a proper guide for the pivot pushed by the extending MSMA material. Due to the wide hysteresis loops, it is key to minimise the friction in nodes as much as possible by choosing appropriate materials such as PTFE. The most frequently applied solution for the spring pretension mechanism is the use of a nut, due to their ease of design and rapid calibration [4,26,27]. Here the spring is always located above the active material in such a way that the moving pivot passes through the hole in the nut, the coil spring being under the nut and surrounding the pivot. However, although such MSMA actuators benefit from a non-complex design and electrically controlled movement, they have several disadvantages, including the necessity of applying a continuous current to maintain the required position, the increase in MSMA temperature due to heat generated by the coils, as well as the reduction in maximum strain caused by spring force [25,34]. For almost the last two decades the Finnish company Adaptamat was the only producer of magnetic shape memory alloys worldwide, but unfortunately in 2014 the company terminated its business. Nevertheless, research in the area of MSMAs continues at ETO Magnetic (Germany), where probes and demonstration actuators are still produced [4,35]. Table 1 presents the most popular sizes and operating modes of actuators containing MSMAs.

In the last two decades, only a few attempts at the application of MSMAs have been presented in the literature, for example in pneumatic proportional valves [36,37] and conspicuous one-stage pneumatic throttle valves [24]. Due to their unique properties, MSMAs can also be used in other mechatronic devices, such as energy harvesters. Varying the MSMA element permeability via alternate compression and elongation by an external force creates a relatively large change in magnetic flux. In [38] a miniature MSMA-based harvesting device with an output power of 0.9 mW/cm^3^ is described, together with a prototype sensor for micro displacement, which uses an *LC* circuit. Elsewhere, the practical usage of MSMA actuators for vibration reduction in a rotor system is described in [39].

**Table 1:** Parameters of MSMA-based actuators presented in the literature.

Actuator Design	MSMA [mm^3^]	Excitation Current [A]	Strain [µm]	Actuator Design	MSMA [mm^3^]	Excitation Current [A]	Strain [µm]
Spring-returned mode				Push—push/push—pull mode			
[29]	2 × 5 × 20	2,2	200	[26]	2 × 3 × 10	5	150
[21,26]	1 × 2.5 × 20	5	400	[32]	3 × 5 × 20	2	1000
[35]	2 × 3 × 15	2	1000	[40]	3 × 5 × 20	2.5	260
[41]	1 × 2.5 × 20	5	400	[31]	3 × 5 × 15	3.5	280
[42]	1 × 2.5 × 20	1.4	900				
[27]	1 × 2.5 × 20	1.2	350				
Mass placed above actuator				Compressive solenoid			
[32,43]	3 × 5 × 20	1	1000	[34]	1 × 2.5 × 20	2	450

## 3. Basics of the MSMA Actuator Design Process

Most MSMA-based actuators presented in the literature can operate within a rather narrow displacement range, typically not greater than 0.7–0.9 mm without load. However, in practice this is reduced to about 0.4 mm due to the return spring, which firstly reduces the maximum displacement and secondly is not able to squeeze the MSMA probe to the same length as it was prior to elongation by the magnetic field. Moreover, in terms of mostly required position control, useful movements cannot be less than 0.3 mm [29]. The main goal of the present paper is thus to present the design, modelling and nonlinear control of an MSMA actuator with repetitive scope of strains in the range from 0 to 1 mm.

**Figure 3:**
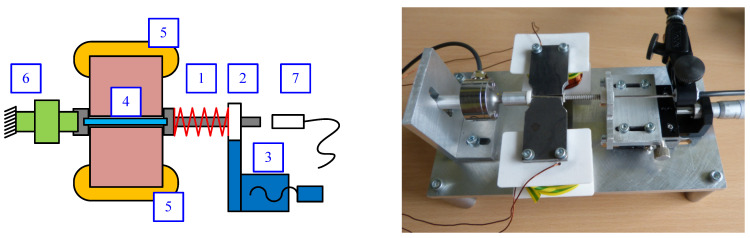
Design of the MSMA actuator used for preliminary studies: 1—coil spring, 2—PTFE sleeve, 3—table, 4—air gap with MSMA, 5—coils, 6—force transducer, 7—displacement transducer.

The first version of the actuator built at Poznan University of Technology (PUT) was designed for the rapid examination of different investigation scenarios (Figure 3). The test stand structure consists of an MSMA sample placed in an air gap (4) of 1 × 2.5 × 20 mm^3^ in a steel S215 magnetic circuit. On one side the actuator stand is equipped with an HBM U9B force transducer (6) with a measuring range of ± 50 N, and on the opposite side with a push rod and coil spring (1) of stiffness 0.43 N/mm. The push rod passes through a PTFE sleeve (2) associated with the table driven by a micrometre screw (3), enabling the compressive force to be adjusted from 0 N up to a blocking force of about 6–7 N. Two coils (5) are located on both sides of the MSMA. Displacement of the actuator is measured by an optical transducer (7).

Even though the first actuator exhibited some imperfections, its testing enabled the preparation of preliminary assumptions regarding design and operating parameters that were used as a base for the design of a new version. Implemented modifications included changes in the shape and material of the magnetic core, the size of the MSMA sample, the shape of the spring, as well as the latter’s type and placement in actuator design. Calculation of the required operating parameters was performed using the following two equations [44]:

(2) $$A_{cs}=\frac{F_{b}}{\sigma_{mag}-2\sigma_{TW}}$$

(3) $$L_{z}=\frac{2y}{\varepsilon_{0}}$$

where: *A_cs_*—cross-sectional area of the MSMA sample (surface perpendicular to the desired direction of deformation), *F_b_*—blocking push force, *y*—desired strain length, *σ_TW_*—twinning stress, *σ_mag_*—maximum value of stress induced by magnetic field (3 MPa [44]), *L_z_*—length of MSMAs element and *ε*_0_—dimensionless free strain expressed as 1–*c*/*a*, where *c, a* are cell dimensions.

In designing the second actuator the following parameters were established as required: *F_b_* = 70 N and minimum repeatable strain *y* = 1 mm. It was assumed that in future research this actuator will also be tested in an electrohydraulic valve, with the assumed parameter values adequate for application with an EPO-45 electromagnet (produced by Fanina), which are widely used in flow control proportional valves. Based on Equations (2) and (3), MSMA probe geometric dimensions of *A_cs_* = 30 mm^2^ and *L_z_* = 32 mm were calculated, resulting in a sample size of 3 × 10 × 32 mm^3^. The probe was commissioned and prepared by Adaptamat Comp.

**Figure 4:**
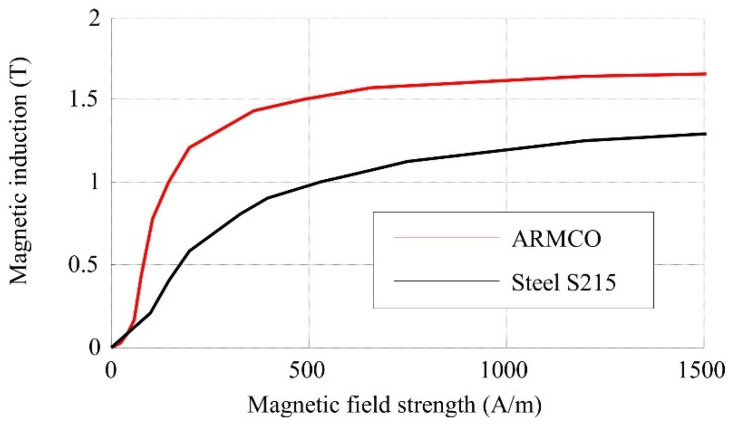
First magnetisation curves for ARMCO iron (steel 04J) and steel S215.

In the second designed at PUT actuator the magnetic core is made of low-carbon (0.035%) steel 04J, which is very similar in chemical composition to pure iron. This soft magnetic steel is distinguished by its very good magnetic properties, similar to those of ARMCO iron and significantly better than those of ordinary construction steel S215, which was used in the first design. The magnetic core parts were precisely annealed by the producer after milling, resulting in secondary recrystallization, which decreases magnetic hysteresis and increases relative permeability, thus maximising magnetic induction. The so-called first magnetisation curves of the magnetic materials considered in the design processes of the MSMA actuators are shown in Figure 4. It is clear from this figure that the ARMCO material is characterised by a higher magnetic induction as a function of magnetic field strength in comparison to steel S215, as well as significantly higher magnetic saturation. Therefore, steel 04J is much more suitable for the application and optimisation of the magnetic core.

**Figure 5:**
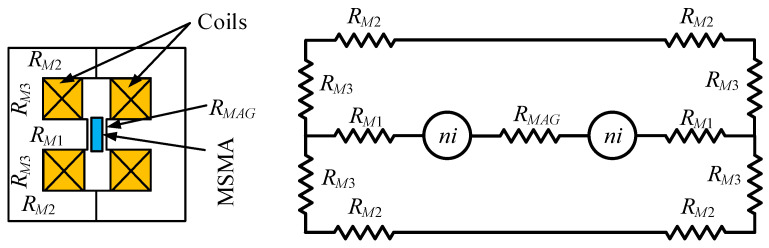
Equivalent magnetic circuit.

The magnetic core consists of two E-shaped parts, with magnetomotive force generated by two identical coils connected in parallel. It was assumed that the magnetic circuit works in linear part of magnetization curve. Based on the basic laws of magnetism, a mathematical model of the magnetic core and coils was elaborated, and the scheme of the equivalent magnetic circuit is presented in Figure 5. The number of coil turns can be calculated using the following equation:

(4) $$n=\frac{\left(2\cdot \left(R_{M1}+R_{M2}+R_{M3}\right)+R_{MAG}\right)\cdot \Phi_{AG}}{i\cdot p}$$

where: *R_M_*_1,_ *R_M_*_2,_ *R_M_*_3_—magnetic reluctances of magnetic core parts (Figure 5.), *R_MAG_*—magnetic reluctance of air gap and in MSMA probe, *Φ_AG_*—magnetic flux in air gap, *i*—coil current, *n*—number of turns and *p*—number of coils.

Magnetic resistances can be calculated as follows:

(5) $$R_{M}=\frac{l}{\mu_{0}\mu_{r}A}$$

where: *l*—length of flux path in core, *A*—cross section of flux path in core and *µ*_0_, *µ_r_*—magnetic permeability of vacuum and relative of path in core.

Considering the magnetisation characteristics of the ARMCO material shown in Figure 4 and the magnetization of the MSMA, it was assumed that the required magnetic induction in air gap *B_AG_* should be 0.7 T, assumed current for single coil was 2 A, thus based on (4) turns were calculated.

**Figure 6:**
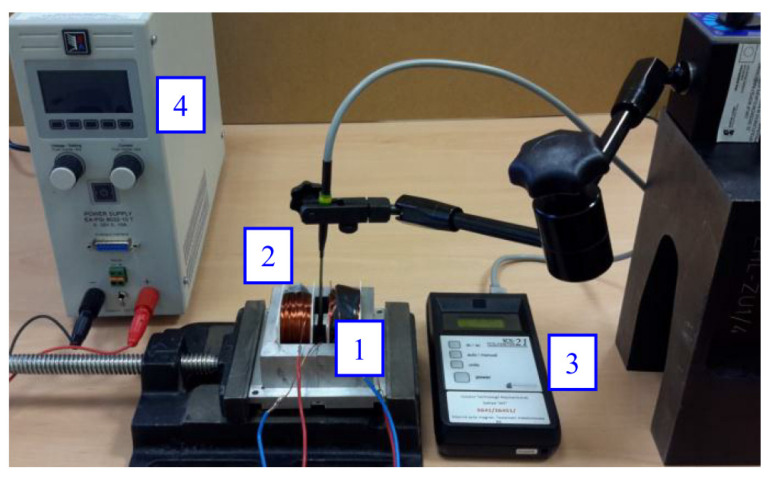
Test stand: 1—examined magnetic circuit, 2—probe, 3—teslameter, 4—DC power supply.

**Figure 7:**
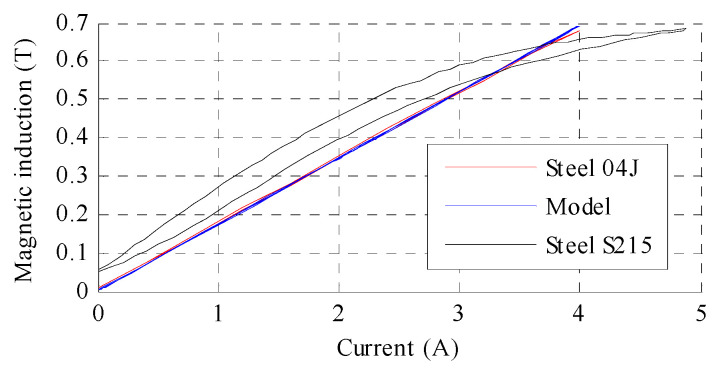
Magnetic induction measured in air gap vs. current for low-carbon steel 04J, steel S215, and model which represents equivalent circuit.

The prepared magnetic core was examined on the dedicated test stand shown in Figure 6 using a Resonance Technology RX-21b teslameter equipped with a Hall effect transverse probe. Variation in magnetic induction (in the air gap) with current was determined and the results are presented in Figure 7. This figure also includes data for steel S215, which exhibited significant magnetisation hysteresis and remanence.

As MSMAs are characterised by low relative permeability, in the calculations they were treated as air (*µ_r_* ≈ 1). Thus, in the presented air gap of width 3.2 mm and area 348 mm^2^ (total clearance between sample and core of 0.2 mm) and for a 2 A excitation current, the calculated magnetic induction is 0.7 T, a value that provides a full range of MSMA deformation. The width of the air gap also plays a key role in coil design, because it has the largest and most significant reluctance. MSMA samples were produced with one side significantly shorter than the other; this shortening should reduce the size of the required magnetic core, especially the coils. In the developed actuator the core is solid, but for improvement of dynamic properties the core should be made of transformer steel sheets (Fe-Si), which would reduce the influence of eddy currents [30].

**Figure 8:**
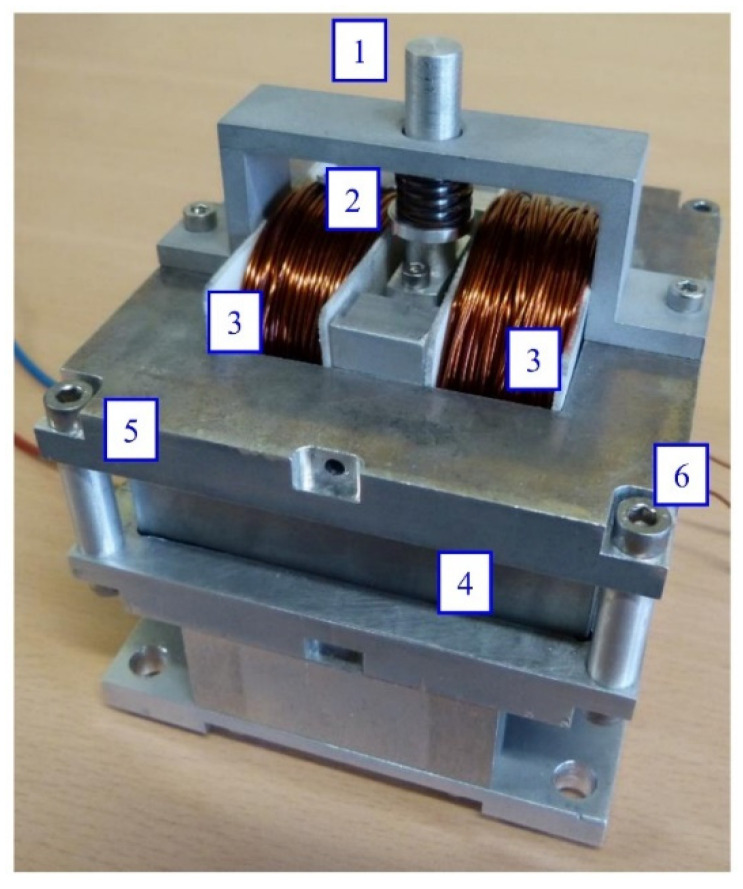
Second actuator design (1)—push rod, (2)—wave spring, (3)—coils, (4)—magnetic pole, (5)—aluminium body, (6)—screws, made of non-magnetic austenitic steel.

The spring used in the MSMA actuator was selected (based on its stiffness) to achieve the desired strain range, as well as being pretensioned adequately in order to obtain the proper compressive force, which depends on the cross-sectional area of the MSMA sample. After conducting a review of those available on the market, it was decided to use a wave spring [45]. This spring possesses the same operating parameters as a classical coil spring, but is 50% smaller in height. The stiffness of the chosen wave spring is 6.93 N/mm, which represents a compromise between its height and mechanical properties. Previous research undertaken by the present authors revealed that an MSMA probe with a cross-sectional area of 1 mm^2^ generates about 2.4 N of force [46]. Because the cross section of the used MSMA sample is 30 mm^2^ (3 × 10 mm^2^), the theoretical maximum output force is equal to about 72 N and thus the spring was pretensioned to about 36 N. Middle value of pretension force provides that maximum displacement is obtained in spring returned mode. A photograph of the assembled actuator is presented in Figure 8.

## 4. Modelling and Control of MSMA Actuator

### 4.1. Static Characteristics Modelling via the Generalised Prandtl-Ishlinskii Model

**Figure 9:**
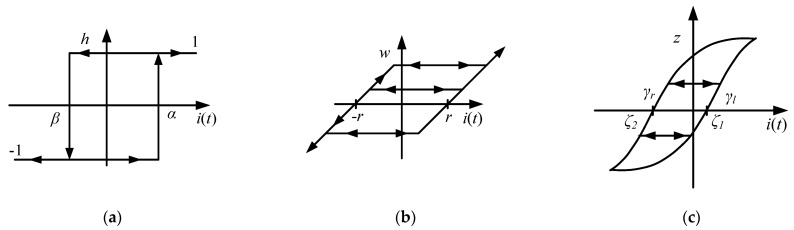
Preisach relay operator (**a**), classical play operator from PIM (**b**) and generalised play operator from GPIM (**c**) for input *i*(*t*).

The MSMA is characterised by saturated hysteresis, which should be compensated in order to obtain good positioning accuracy. The developed approaches can be divided into physics-based models [47] and phenomenological models. The first one uses hysteretic behavior and principles of physics effects. The example is the model, developed for hysteresis of ferromagnetic materials [48]. Nowadays phenomenological models are most commonly used and applied. There are operator-based models, such as: Preisach [49], Krasnosel’skii-Pokrovkii [50], Prandtl–Ishlinskii [51]. The earliest model of hysteresis is that developed by Preisach (1935), in which parallel connections of independent relay hysterons are applied. The hysteron input is a piecewise continuous monotonic function and its output *h* varies between 1 and −1 (Figure 9a). This model uses an infinite set of elementary hysteresis operators, which are similar to relay characteristics.

A modification of the Preisach model is proposed in [52]. Both the generalized Prandtl-Ishlinskii model and a classical Prandtl-Ishlinskii model are well-known phenomenological hysteresis models [53]. A clear mathematical description of different models of hysteresis such as: Preisach, Ishlinskii, Krasnoselskii and Duhem–Madelung are presented by Macki et al. in [54]. The properties and the comparison of these models are also included. Hassani et al. in [55] presented the survey on hysteresis modeling, including such various models as Preisach, Krasnosel’skii–Pokrovskii, Prandtl–Ishlinskii, Maxwell-Slip, Bouc–Wen and Duhem. In the paper also their applications in control and identification are presented. A similar solution is the Maxwell-slip model, which is designed for modeling of friction. It can be adapted for smart actuators modelling [56]. Another group of models is based on differential equations. The examples are Duhem model [57], Backlash-like [58] and Bouc-Wen. The last model was introduced by Bouc [59]. Wen extended this formula in paper [60].

In recent years, different artificial neural networks have also been successfully used in modelling of different actuators’ hysteresis. Yu et al. in [61] proposed the rate-dependent hysteresis NARMAX model based on a diagonal recurrent neural network. As the variable function of the NARMAX model, the adopted play operator is used. The experimental investigations show that the proposed model has a very good modeling precision. The proposed approach may enable the broadening of the application based on MSMA actuators in micro/nano devices. The investigations on compensation of MSMA-based actuator hysteresis is described in [62]. To this end, a feed-forward neural network-based nonlinear autoregressive moving average with exogenous inputs model is applied. The modelling and control methods are investigated experimentally using an MSMA-based actuator. The results show that the proposed solution allows an accurate description of the hysteresis of the actuator and the developed compensator can effectively reduce the impact of hysteresis on the performance of the MSMA-based actuator.

In the oft-used Prandtl-Ishlinskii (PI) model, the relay hysterons are replaced by a play operator *F_r_*, which can be represented by backlash (a well-known phenomenon in mechanical systems), with the width of the backlash hysteresis defined by two thresholds *r* (Figure 9b). The generalised PI model (GPIM) is more suitable for the modelling of constant non-linear hysteresis. Detailed descriptions of hysteresis modelling techniques involving this model can be found in [23,54].

The mathematical function describing the model input, here the current signal *i*(*t*), must satisfy the condition of monotonicity in each subinterval [*t_q_*, *t_q_*_+1_] with *t_q_* < *t* ≤ *t_q_*_+1_ and 0 ≤ *q* ≤ *N*−1. A mathematical description of operator output *F_r_* for *t*_0_ = 0, *t*_0_ < *t*_1_ < *t*_2_ < … < *t_N_* = *t_E_*, can be formulated as

(6) $$F_{r}\left(i(t)\right)=\{\begin{array}{c} \max\left(i(t)-r,\;w(t_{q})\right),\\ \min\left(i(t)+r,\;w(t_{q})\right),\\ w(t_{q}), \end{array}\;\begin{array}{c} for\\ for\\ for \end{array}\;\begin{array}{c} i(t)>i(t_{q})\\ i(t)<i(t_{q})\\ i(t)=i(t_{q}) \end{array}$$

where: *F_r_*—current value of play operator in each time subinterval, *w*(*t_q_*)—play operator output in previous time moment, *i*(*t*)—monotonous input signal, *i*(*t_q_*)—value of input signal in previous time moment, and *r*—threshold value. The operator must satisfy the initial condition expressed as *F_r_*(*i*(0)) = *w*(0).

The input-output relationship *y_p_* in the Prandtl-Ishlinskii model is indicated by

(7) $$y_{p}\left(t\right)=\int_{0}^{R}p(r)\cdot F_{r}\left(\left(i\right)t\right)\cdot dr$$

where: *p*(*r*)—density function, which is a representation of the influence of each operator on the final shape of hysteresis and *R*—upper integration limit.

In order to model the asymmetric and saturated shapes of the hysteresis characterising the MSMA actuators, the play operator described by Equation (6) was modified, becoming the generalised operator expressed as *G_r_* (Figure 9b). In the *G_r_* operator, the increasing curve (function) *γ_l_* and the decreasing curve *γ_r_* are so-called envelope functions and must be continuous. The most suitable function with which to model both major and minor hysteresis loops in MSMA actuators is the hyperbolic tangent. The play operator *G_r_* for the generalised model must fulfil the same conditions as play operator *F_r_*. The mathematical formulation of *G_r_* is described by the following equation:

(8) $$G_{r}\left(i\left(t\right)\right)=\{\begin{array}{c} \max\left(\gamma_{l}\left(t\right)-r,\;z\left(t_{q}\right)\right),\\ \min\left(\gamma_{r}\left(t\right)+r,\;z\left(t_{q}\right)\right),\\ z\left(t_{q}\right), \end{array}\begin{array}{c} \;for\\ \;for\\ \;for \end{array}\;\;\begin{array}{c} i\left(t\right)>i\left(t_{q}\right)\\ i\left(t\right)<i\left(t_{q}\right)\\ i\left(t\right)=i\left(t_{q}\right) \end{array}$$

where: *G_r_*—current value of play operator in each time subinterval, *z*(*t_q_*)—play operator output in previous time moment, *i*(*t*)—monotonous input signal, *i*(*t_q_*)—value of input signal in previous time moment, and *r*—threshold value.

*G_r_* must also meet the same initial condition as the classical operator, i.e., *G_r_*(*i*(0)) = *z*(0). In the hysteresis model, each play operator can be weighted by the density function *p*(*r*), which helps to match the model to the measured hysteresis and is described by Equation (9) as follows [23,47]:

(9) $$p\left(r_{j}\right)=\rho \cdot e^{-\tau \cdot r_{j}}$$

where: *r_j_* = *α·j* (*j* = 0, 1, …, *m*).

The total number of used operators *j* is equal to *m* + 1. Parameters *α*, *ρ* (always positive) and *τ* allow for the precise description of the density function for the *j*-th operator. The output of the model described above is calculated as

(10) $$y_{p\gamma }\left(t\right)=\int_{0}^{R}p\left(r\right)\cdot G_{r}^{}\left(\left(i\right)t\right)\cdot dr$$

However, for practical implementation in a control scenario this integral can be expressed as a superposition of a finite number of generalised play operators, where each one is weighted by a unique value of density function *p*(*r_j_*):

(11) $$y_{p\gamma }(t)=\sum_{j=0}^{m}p\left(r_{j}\right)\cdot G_{r_{j}}\left(\left(i\right)t\right)$$

This equation can be simply implemented in a computer-based controller.

Hysteresis loops in the MSMA actuator are characterised by saturation. As their shape is very similar to that of the hyperbolic tangent function, this function can be used to describe the decreasing and increasing slopes of the hysteresis envelope curves via the following equations:

(12) $$\gamma_{l}=a_{0}\tanh\left(a_{1}i+a_{2}\right)+a_{3}$$

(13) $$\gamma_{r}=b_{0}\tanh\left(b_{1}i+b_{2}\right)+b_{3}$$

where: *a*_0_, *a*_1_, *a*_2_, *a*_3_, *b*_0_, *b*_1_, *b*_2_, *b*_3_ are the parameters of the hyperbolic tangent functions.

### 4.2. Inverse of Generalised Prandtl-Ishlinskii Model

The application of an inversed model can be used for the compensation of actuator hysteresis and thus can significantly improve its properties [48]. It was proven in a paper [23] that the generalised Prandtl-Ishlinskii model has an analytical inversion (based on the initial loading curve concept) meeting one condition, that the envelope function must also be analytically invertible. Based on the aforementioned paper the inversed function *y_pγ_^−^*^1^ can be written

(14) $$y_{P\gamma }^{-1}\left(t\right)=\{\begin{array}{c} \begin{array}{ccc} \gamma_{l}^{-1}\left(\sum_{j=0}^{n}\hat{p}\left(r_{j}\right)F_{{\hat{r}}_{j}}\left(\left(y_{l}\right)t\right)\right) & for & \frac{dy_{r}}{dt}\geq 0 \end{array}\\ \begin{array}{ccc} \gamma_{r}^{-1}\left(\sum_{j=0}^{n}\hat{p}\left(r_{j}\right)F_{{\hat{r}}_{j}}\left(\left(y_{r}\right)t\right)\right) & for & \frac{dy_{r}}{dt}<0 \end{array} \end{array}$$

where: *y_r_*—position reference for the actuator control system, *γ_l_*^−1^, *γ_r_*^−1^—inverse envelop functions, $\hat{p}\left(r_{j}\right)$—density function for inverse model, $F_{{\overset{⌢}{r}}_{j}}$—play operator for redefined threshold ${\overset{⌢}{r}}_{j}$.

This model utilises a new set of parameters obtained via the following equations:

(15) $${\hat{r}}_{j}=\sum_{k=0}^{j}p_{k}\left(r_{j}-r_{k}\right)$$

Density function defined for *k* = 0

(16) $${\hat{p}}_{0}=\frac{1}{p_{0}}$$

and interval where k∈〈1;j〉

(17) $$\hat{p}\left(r_{j}\right)=\frac{1}{\sum_{k=0}^{j}p\left(r_{j}\right)-\sum_{k=0}^{j-1}p(r_{j})}$$

For example, the inversion of Equation (12) necessary in the compensation model is formulated as follows:

(18) $$\gamma_{l}^{-1}\left(i\right)=\frac{\left(\tanh^{-1}\cdot \left(\frac{i-a_{3}}{a_{0}}\right)-a_{2}\right)}{a_{1}}$$

### 4.3. Actuator Dynamics Model

**Figure 10:**
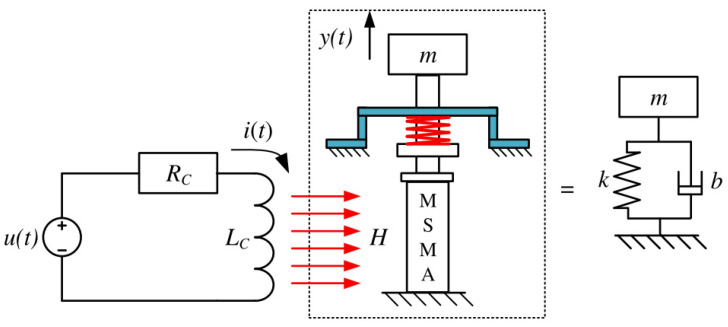
Schematic representation of the electromechanical structure of the MSMA actuator, where: *u*(*t*)—supply voltage, *R_c_*—coil resistance, *L_c_*—coil inductance, *i*(*t*)—current, *y*(*t*)—mass displacement, *H*—magnetic field strength, *m*—mass, *b*—resultant damping coefficient, and *k*—resultant spring constant.

The MSMA actuator is an electromechanical device, the structure of which is presented schematically in Figure 10; this structure is a simplified representation for dynamic modelling purposes.

The electric part consists of a coil connected serially to a resistor, as described by the following differential equation:

(19) $$u\left(t\right)=R_{C}i\left(t\right)+L_{C}\frac{di\left(t\right)}{dt}$$

The transfer function of the actuator’s electrical part can thus be written as follows:

(20) $$G_{c}\left(s\right)=\frac{I(s)}{U(s)}=\frac{1}{L_{C}s+R_{C}}$$

The current flow generates the magnetic field strength, which is proportional to this current and to the number of coil turns. Magnetic flux in the air gap acts on the MSMA, with the particles in the crystal lattice changing their orientation and thus the output force *F_re_* is created.

Assuming linearity, the dynamics of the actuator’s mechanical part can be described using the force equilibrium formulated as the following differential equation:

(21) $$F_{re}\left(t\right)=m\ddot{y}\left(t\right)+b\dot{y}\left(t\right)+ky\left(t\right)$$

where: *F_re_*—force generated by magnetically induced reorientation of crystal lattice, *m*—mass of moving parts, *b*—mechanical damping constant and *k*—spring constant of system.

After Laplace transformation, this equation can be rewritten as the transfer function of the mechanical part:

(22) $$G_{m}\left(s\right)=\frac{Y(s)}{F_{re}(s)}=\frac{1}{s^{2}m+sb+k}$$

**Figure 11:**
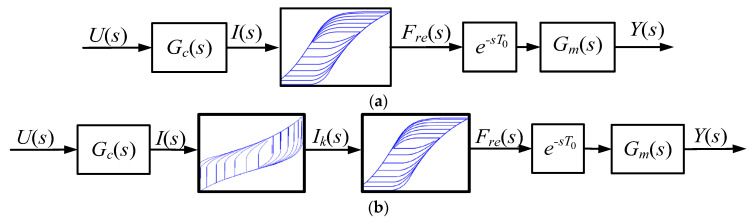
MSMA actuator model structure: (**a**) classical approach, (**b**) with hysteresis compensation.

Hysteresis is caused by nonlinear dependence between input current and the force that generates movement. The model of the actuator with hysteresis is shown in Figure 11a. As preliminary laboratory research revealed that the above-described MSMA actuator is characterised by time delay, the model also includes a delay block. To compensate for the hysteresis, an additional element was applied in the form of the inversed GPI (IGPI) model of hysteresis. Thus, it can be assumed that the relationship between input current and output force is linear and can be expressed by transfer function (16), with gain factor *k_F_* (Figure 11b).

Based on Figure 11b and omitting the nonlinearities, the transfer function of the linearized actuator is expressed as

(23) $$G_{MSMA}\left(s\right)=\frac{k_{F}\cdot e^{-sT_{0}}}{s^{3}L_{c}m+s^{2}(bL_{c}+mR_{c})+s\left(kL_{c}+R_{c}b\right)+R_{c}k}$$

## 5. Test Stand

**Figure 12:**
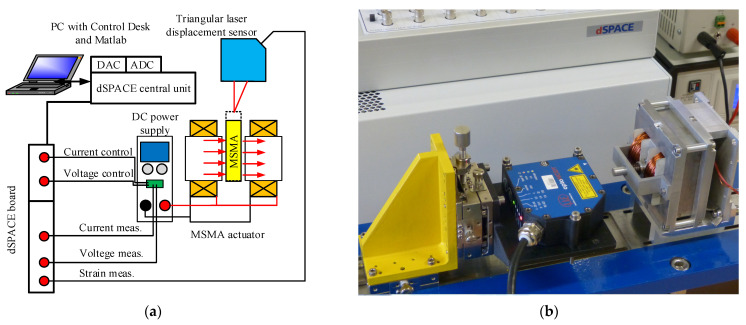
Test stand schematic diagram (**a**) and assembled set up (**b**).

In order to analyse the performance of the revised MSMA actuator, a special test stand was designed and built, with the stand schematic and photograph presented in Figure 12. The entire instrumentation and actuator setup was placed on a thick, solid steel plate (1) of weight around 20 kg, providing necessary stiffness and stability. The plate itself was also assembled on levelling elements with damping elastomer. This was carried out because during the earlier investigations undertaken using the stand shown in Figure 3, the strong influence of external vibration on the obtained results was recorded, especially during dynamics testing. Rapid calibration of the test stand elements was provided by table displacement driven by micrometre screws (2). For actuator position measurements, an ILD-1700-2 triangular laser sensor (3) was used. A high accuracy DC power supply (4) source was employed to supply the coil (*u* = 0 ÷ 32 V, *i* = 0 ÷ 10 A), controlled by a 0–10 V analogue input generated by a dSPACE output card. Experiments were performed using the dSPACE platform with AD and DA converters (5), with the examined actuator indicated by the number 6.

## 6. Hysteresis Compensation and MSMA Actuator Control

**Figure 13:**
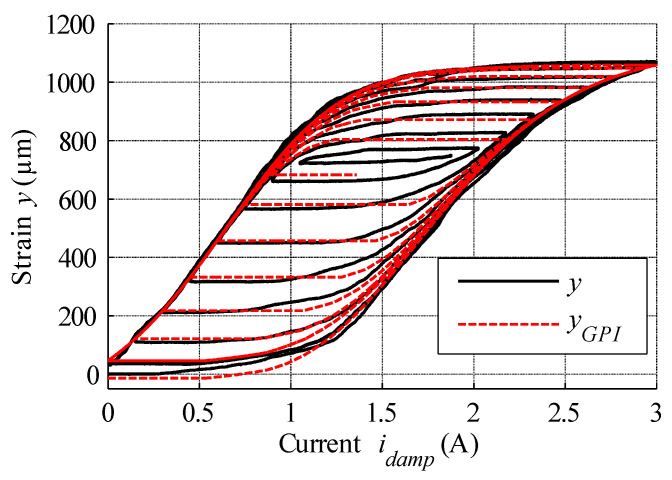
Comparison of hysteresis curves obtained via measurement and simulation for damped sine input (black curve is measured, red curve is output of simulation model).

**Figure 14:**
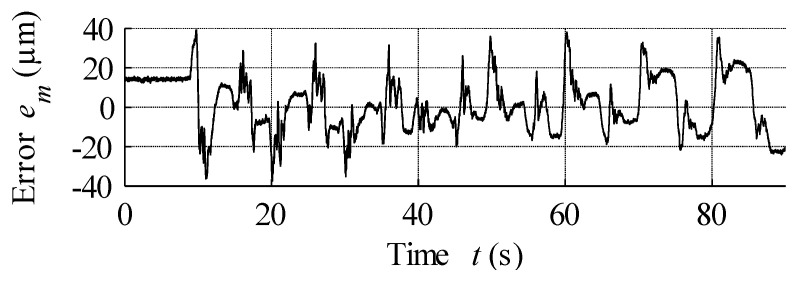
Modelling error between curves presented in Figure 13.

During the first examination of the designed actuator, its static characteristics were measured. To this end, the asymptotically damped sinusoidal voltage was supplied to the power supply input, which then amplified this signal and passed it on to the coils, thereby ensuring the registration of both major and minor hysteresis loops. The set of recorded actuator position changes is presented in Figure 13. These data were then used for the estimation of necessary hysteresis modelling parameters via the nonlinear least squares method, as summarised in Table 2. These parameters were then used in the GPI model of hysteresis, with the simulation results displayed in Figure 13. Finally, the modelling error expressed as the difference between measured data and model output is presented in Figure 14.

Analysis of Figure 14 reveals the presence of bias up to the 9th second, with the model output for a 0 A input being non-0 and constant. The reason for this result is the fact that it is not possible to obtain 0 output from the model. An analysis of modelling errors is summarised in Table 3. As can be seen from this table, the maximum values of modelling error are significantly lower than those presented elsewhere in the literature for studies employing the modified PI model. For example, the maximum absolute error recorded here was 3.7%, much lower than the 11.2% reported in [49] and the value of 14.5% reported in [50]. Although the application of the Krasnosel’skii-Pokrovskii model in [29,51] provided better results than the modified PI, the maximum absolute error was still 8.16%, a value again significantly larger than that reported in the present paper.

**Figure 15:**
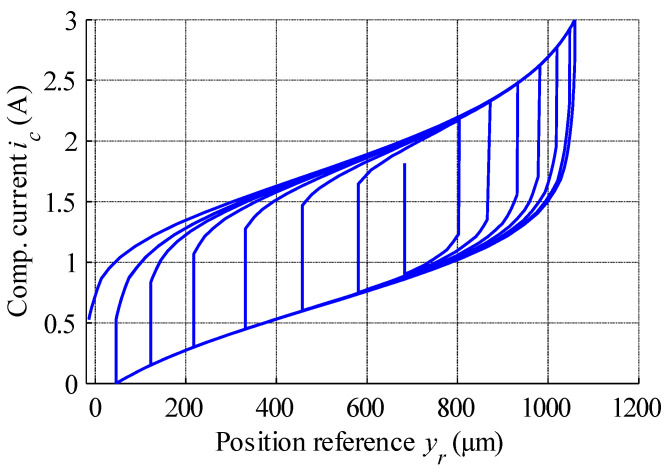
IGPI model output showing the relationship between position reference and compensation current.

The inverse GPI model was then prepared based on the estimated parameters of the revised MSMA actuator, and the simulation results obtained using the inverse model are shown in Figure 15. The inverse model can be applied in a real-time control system as a compensator of hysteresis nonlinearity, a block diagram of which was shown above in Figure 11b.

**Figure 16:**
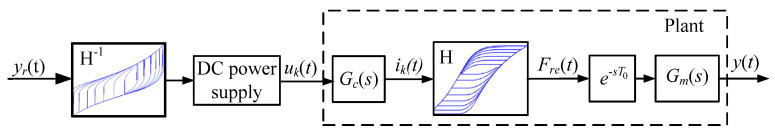
Block diagram of open-loop control system.

Figure 16 shows the scheme of the open-loop control system used for the second MSMA actuator, based on the above inverse GPI hysteresis model. System input is the position reference signal, which is described by the sum of sine and cosine trigonometric functions as expressed in the following equation:

(24) $$y_{r}\left(t\right)=250\cdot \sin\left(1.4\pi \cdot t\right)+250\cos\left(2\pi \cdot t\right)+500$$

**Figure 17:**
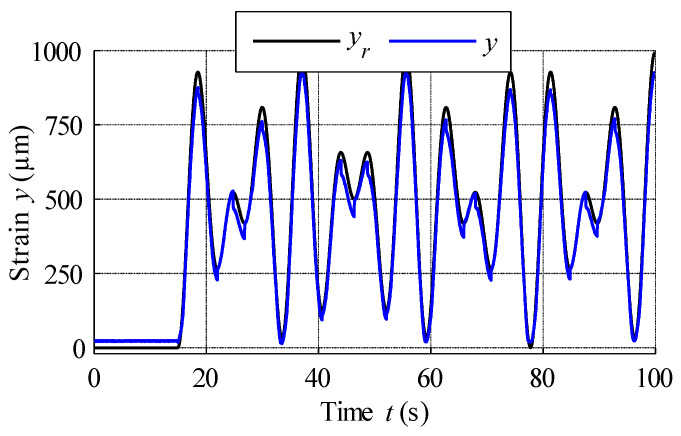
Open-loop control with compensator placed in series in the control loop.

**Figure 18:**
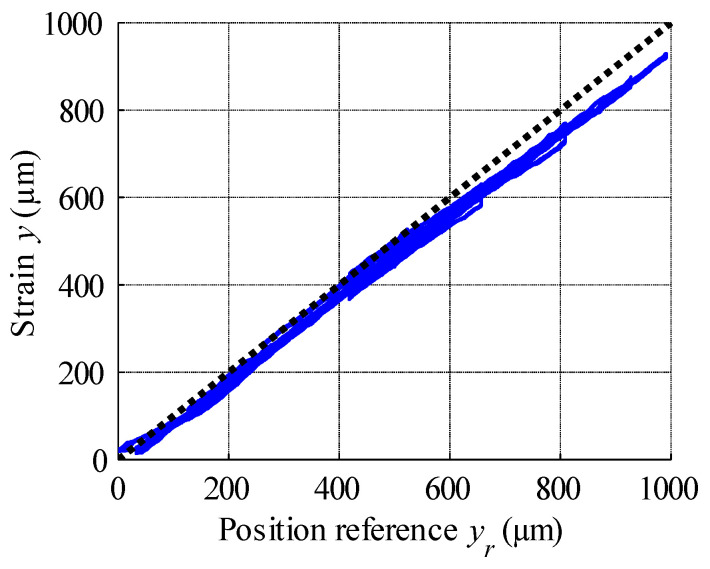
Linearised characteristics of the MSMA actuator.

**Figure 19:**
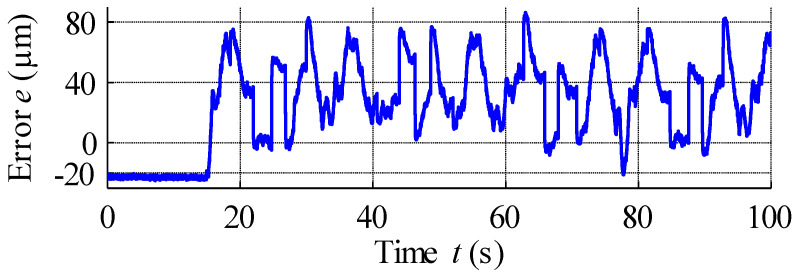
Compensation error.

**Figure 20:**
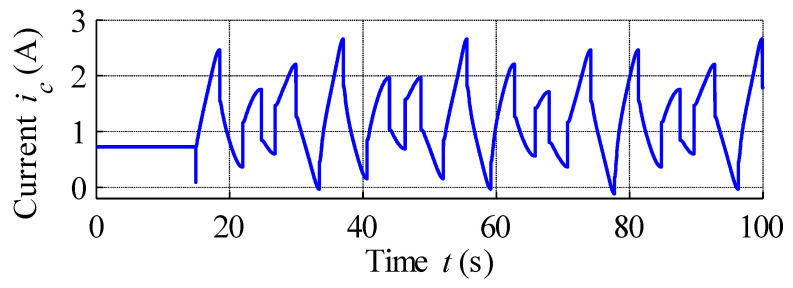
Nonlinear current control signal.

The result of open-loop compensation is presented in Figure 17, which shows that the application of reference signal tracking provided much better results compared to the control scheme without hysteresis compensation. The position reference vs. measured strain curve plotted in Figure 18 shows a linearization effect, with significant hysteresis reduction. Analysis of Figure 19, which displays the variation in the compensation error over time, reveals constant errors at the beginning of the actuator operation, likely the result of errors arising from the hysteresis modelling process. Here error is defined as the difference between reference signal and measured strain, and was positive throughout almost the entire experiment, with its magnitude increasing with the magnitude of the position reference signal. Complex analysis of compensation errors is presented in Table 4, and the output of the hysteresis model, or control effort, presented in Figure 20.

**Figure 21:**
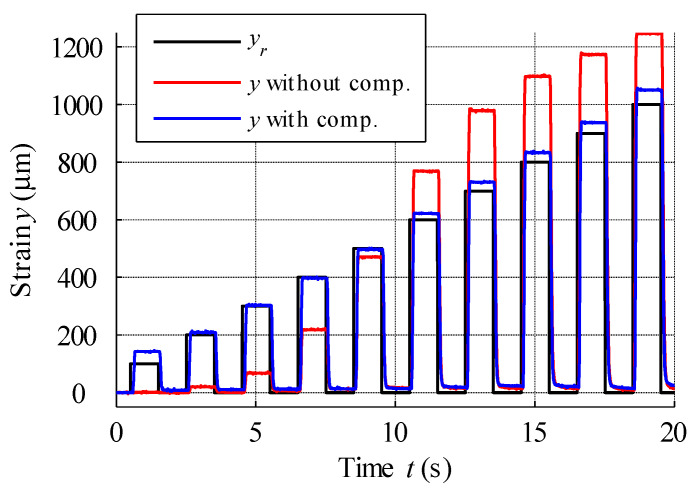
Open-loop control for a series of rectangular position references with increasing amplitude.

**Figure 22:**
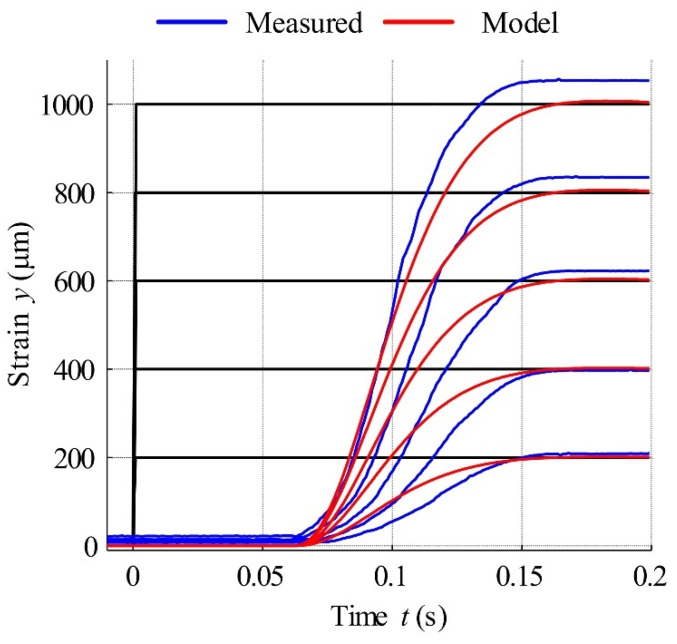
Step responses of actuator and simulation model described by Equation (23).

In the next test, the reference signal was analysed as a series of rectangular steps, with an amplitude increase of 100 µm at each step. Figure 21 displays this signal response for the open-loop control of actuators with and without hysteresis compensation. Analysis of this figure reveals a clearly visible effect of twinning stress in the actuator controlled without compensation. In this case, despite the increase in excitation current, response signals of up to 400 µm were disrupted by hysteresis and the mechanical friction of moving parts. For input signal steps greater than 600 µm in amplitude, the actuator output signal is distinguished by high overshoot. The step responses of the dynamic simulation model built using Equation (19) and the MSMA actuator with hysteresis compensation are presented in Figure 22, with actuator parameters as follows: coil inductance 13 mH, coil resistance 1.6 Ω, mass of moving parts 0.02026 kg, damping coefficient 1.8 Ns/mm, spring constant 62.7 N/mm and force gain 25 N/A. Thus, the actuator gain was equal to 0.249 mm/V. The response of the MSMA actuator was characterised by a time delay of 65 ms, with the dynamic model better covering higher magnitude steps.

### PID Control

**Figure 23:**
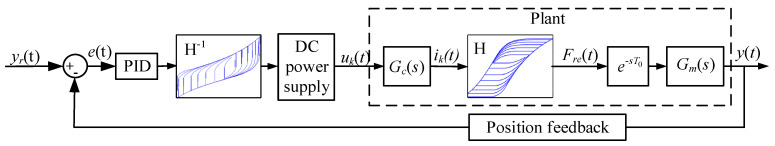
Block diagram of closed-loop control system with direct hysteresis compensation by the inverse model.

The closed-loop control system was then examined based on the developed dynamic model, as shown in Figure 23; PID controller gains were established using the Matlab Optimisation Toolbox (Table 5) [52,53].

**Figure 24:**
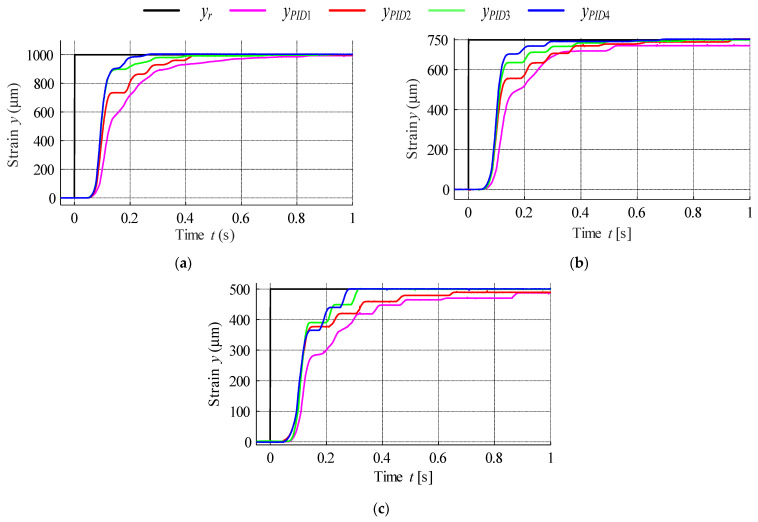
Actuator step response for position references: (**a**) 1000 µm, (**b**) 750 µm and (**c**) 500 µm.

Actuator response was investigated for three position reference steps (500 µm, 750 µm, 1000 µm), with the results presented in Figure 24; in Table 6 the rise time was also specified for each response.

Integral absolute errors *IAE* were calculated via the following formula and the results presented in Table 7:

(25) $$IAE=\frac{1}{N}\sum_{k=1}^{N}\left|y_{r}\left(t_{k}\right)-y\left(t_{k}\right)\right|$$

**Figure 25:**
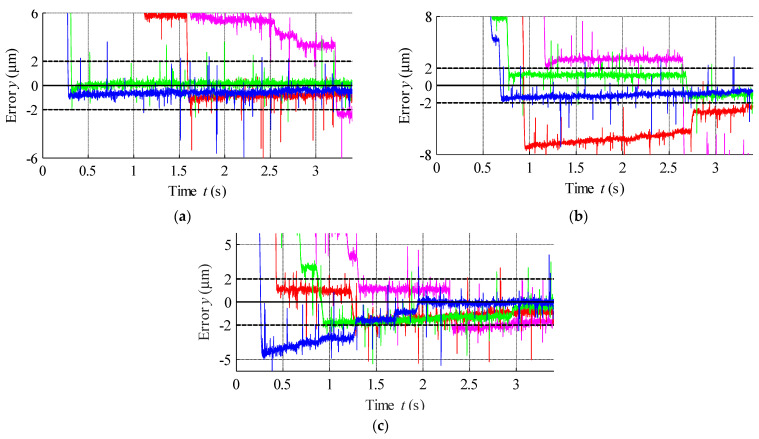
Positioning error for the step responses presented in Figure 24: (**a**) 1000 µm, (**b**) 750 µm and (**c**) 500 µm.

The positioning error for each step response was then plotted in Figure 25 based on the measured data presented in Figure 24. According to the obtained results the error band can be established as ±2 µm.

## 7. Conclusions

This paper has provided a description of the design, modelling and control of two MSMA actuators, summarising four years’ research performed at Poznan University of Technology in the Division of Mechatronic Devices. The presented results indicate that MSMA actuators are still worth considering in positioning systems, with the control of nonlinear objects possible through the use of adequate compensation techniques and strategies. The chosen modelling technique provided very satisfactory and promising results, achieving a lower hysteresis modelling error than reported elsewhere.

During the design process, a maximum current of 4 A was assumed, but considering the relative permeability of MSMA, which increases with deformation, a lower excitation current will be needed to generate the required flux density. Maximum elongation was here obtained for a current of 3 A, 25% less than in preliminary calculations. In the developed actuators, the coil size is determined by heat emission and current density in wires. Research into MSMAs will continue, with a next generation actuator in development based on biased magnetic flux and energy harvesting.

The most influencing limitations and disadvantages of MSMA-based actuators are: hysteresis, relatively low dynamics and the need to use springs to return the transducer to its original position. Nevertheless, they can be successfully used in many practical devices.

The results obtained in the paper show that the MSMA-based actuators are very interesting for their application in design of a new class of high accuracy, innovative positioning transducers. In the future, we plan to apply MSMA in the electro pneumatic or electrohydraulic valves and to design a novel harvesting device. Also the application of MSMA in special brakes will be considered.

## Figures and Tables

**Table 2:** Estimated GPIM parameters.

GPIM Parameters		
Model order *m*	10	
Operator scope	11 (0, 1…10)	
Envelope functions *γ*	*a*_0_ = 1.906	*b*_0_ = 1.9931
	*a*_1_ = 1.1530	*b*_1_ = 1.2839
	*a*_2_*=* −1.9515	*b*_2_*=* −0.8924
	*a*_3_ = 1.5358	*b*_3_ = 1.2934
Density function parameters *p*(*r*)	*α =* 0.1255	
	*ρ =* 110.5488	
	*τ =* 2.9377	

**Table 3:** Analysis of GPI errors.

Error Type/Error Value	(µm)	%
MSMA actuator strain	1074.5	100
Max. positive error	39.73	3.70
Max. negative error	−37.45	3.49
Max. absolute error	39.73	3.70
Mean error	2.58	0.24
Mean absolute error	11.71	1.09
RMSE	13.9	1.29

**Table 4:** Compensation errors.

	Reference *y_r_*(*t*)	
	(µm)	%
MSMA actuator strain	1000	100
Max. positive error	86.37	8.64
Max. negative error	−24.98	2.50
Max. positive error + |Max. negative error|	111.35	11.14
Max. absolute error	86.37	8.64
Mean error	26.25	2.63
Mean absolute error	33.75	3.38
RMSE	39.62	3.96

**Table 5:** PID controller gains.

Controller	Gains		
	kp	ki	kd
PID1	0.3	6	0.01
PID2	0.5	8	0.01
PID3	0.55	10	0.015
PID4	0.57	11,3	0.02

**Table 6:** Rise time.

Rise Time *t_u_* [ms] for *y_r_* ±5%						
Controller	Position reference *y_r_* [µm]					
	500	+25	750	+37.5	1000	+50
		−25		−37.5		−50
PID_1_	770		510		480	
PID_2_	470		370		340	
PID_3_	301		295		250	
PID_4_	230		202		180	

**Table 7:** Integral absolute error.

	Integral Absolute Error		
Controller	Position reference *y_r_* [µm]		
	500	750	1000
PID_1_	126.3	154.9	187.1
PID_2_	92.4	126.3	142.8
PID_3_	70.25	99.02	116.9
PID_4_	72.35	89.44	108.4

## Data Availability

Data are available on request.
